# Mental Health and Its Predictors during the Early Months of the COVID-19 Pandemic Experience in the United States

**DOI:** 10.3390/ijerph17176315

**Published:** 2020-08-31

**Authors:** Yanmengqian Zhou, Erina L. MacGeorge, Jessica Gall Myrick

**Affiliations:** 1 Department of Communication Arts and Sciences, Pennsylvania State University, 234 Sparks Building, University Park, PA 16802, USA; elm26@psu.edu; 2 Donald P. Bellisario College of Communications, Pennsylvania State University, 201 Carnegie Building, University Park, PA 16802, USA; jgm43@psu.edu

**Keywords:** COVID-19, mental health, Depression, Anxiety and Stress Scale (DASS-21), posttraumatic growth, longitudinal design

## Abstract

To date, there has been relatively little published research on the mental health impacts of COVID-19 for the general public at the beginning of the U.S.’ experience of the pandemic, or the factors associated with stress, anxiety, depression, and post-traumatic growth during this time. The current study provides a longitudinal examination of the predictors of self-reported stress, anxiety, depression, and post-traumatic growth for U.S. residents between April and May, 2020, including the influence of demographic, psychosocial, and behavioral factors on these outcomes. The findings indicate that, generally, the early months of the U.S. COVID-19 experience were characterized by a modest negative impact on mental health. Younger adults, people with pre-existing health conditions, and those experiencing greater perceived risk, higher levels of rumination, higher levels of co-rumination, greater social strain, or less social support reported worse mental health. Positive mental health was associated with the adoption of coping strategies, especially those that were forward-looking, and with greater adherence to national health-protection guidelines. The findings are discussed with regard to the current status of health-protective measures and mental health in the U.S., especially as these impact future management of the on-going pandemic.

## 1. Introduction

In mid-March of 2020, the World Health Organization declared COVID-19 to be a global pandemic and, in the U.S., a national emergency was declared. Starting in this same timeframe, health-protective measures were put into effect by government bodies at multiple levels across the country. Although variable in onset, duration, and stringency, these measures included school, business, and government closings, social distancing and quarantine requirements, and face-covering policies (this latter largely after April 3rd, based on revised guidance from the Centers for Disease Control and Prevention (CDC) [1]). Thus, as illness and death toll climbed through March and April, so too did the public’s need to cope with significant “secondary” stressors, such as temporary or permanent job loss and associated financial difficulties, challenges obtaining food and other material goods, educating and caring for children at home, physical separation from family and friends, and the discomforts (both physical and psychological) associated with wearing masks and other face coverings.

As the pandemic and public health measures escalated, experts in various fields quickly began to note the probability of negative impact on mental health in the general public and the need for additional or improved mental health services [2,3,4,5]). Indeed, the probability that COVID-19 will have at least short-term negative effects on mental health among the U.S. general public is suggested by research from prior pandemics, especially SARS-CoV-1 [6] and by recently-published studies (for a review, see [7]), especially those conducted with Chinese citizens who first experienced the pandemic’s outbreak and their government’s extensive measures to prevent the disease from spreading [8,9,10,11,12,13]. These studies document detrimental effects of COVID-19 on depression, anxiety, post-traumatic stress, and sleep quality. Similar negative effects have also been reported from other countries [14,15,16,17], along with increases in smoking and alcohol consumption, and decreased physical activity [16]. These studies have also identified various factors that either make mental health issues more likely or protect against them. For example, in a very large (*N* = 52,730) cross-sectional Chinese study conducted in January and early February of 2020, Qiu et al. [11] found that psychological distress was higher for women, young adults (18–30) and the elderly (over 60), migrant workers, people who lived closer to the center of the epidemic in China, and those for whom healthcare access was problematic. A longitudinal study by Wang et al. [12] conducted between January 31st and March 1st identified confidence in doctors, satisfaction with health information, and adopting precautionary measures (e.g., hand-washing) as factors protective against stress, anxiety, and depression.

To date, there have been few empirical research reports on the mental health conditions of the general public at the beginning of the US experience of the COVID-19 pandemic, or the factors that were associated with mental health at that time. Understanding the shifts in the public’s mental health status during the pandemic and the factors that may be influencing changes in mental health is relevant to understanding public responses to the on-going pandemic, planning for subsequent management of COVID-19, and preparing for future pandemics. Accordingly, the current study provides a longitudinal examination of self-reported stress, anxiety, depression, and post-traumatic growth as experienced by U.S. residents in April and May, 2020. The goal of this study was to gain insights into the influence of various demographic, psychosocial, and behavioral factors that may shape changes in these mental health outcomes across the course of a pandemic. Psychosocial factors examined included perceived health and financial risks, rumination and co-rumination about the pandemic, social support, and social strain. Behavioral factors measured included adherence to national public health guidelines (issued by the Centers for Disease Control and Prevention (CDC) on March 16) and coping strategies. Demographic factors were gender, age, race, ethnicity, education, region of residence, political party, political orientation (liberal-conservative), employment status, pre-existing health conditions, experience of COVID-19-consistent symptoms, and COVID-19 testing.

## 2. Method

### 2.1. Study Design and Participants

Participants aged 18 to 90 were recruited through Qualtrics.com to take part in a series of online surveys. Qualtrics recruits its panelists from various online sources (e.g., website intercept recruitment, member referrals, targeted emails, customer loyalty portals, and social media). Qualtrics panels are frequently used in health-related research to obtain representative samples, and studies show that samples obtained through Qualtrics have demographic attributes that align (with approximately 10% variation) with the 2010 Census data [18]. For this study, potential participants were sent an email invitation by Qualtrics with a link to the questionnaire and information about participation incentives (typically worth $4–$5 per survey). Once the participants provided informed consent, they were directed to complete the study survey. In total, 1021 participants were recruited at Wave 1, 633 continued at Wave 2, and 442 remained at Wave 3 (56.71% attrition between Wave 1 and Wave 3). As the study was conducted online, we could not control participants’ environment or level of engagement with the study. We did, however, utilize mechanisms available through Qualtrics for quality control: the median duration of study completion was computed during a soft launch, and subsequent participants who spent less than one-third of that time on the study were dropped. Wave 1 took place on April 20, Wave 2 between May 4 and May 8, and Wave 3 between May 18 and May 22, with a two-week lag between initiation of each wave. All procedures were approved by the Institutional Review Board at the Pennsylvania State University (STUDY00014927) and participants provided informed consent prior to beginning the surveys.

Details of the demographic characteristics of the Wave 1 sample are shown in Table 1. The average age of participants was slightly over 45 years old. The sample was divided near-evenly between males and females and identified primarily as White (75%) or Black (11%), with less representation from other racial groups. Almost 10% were of Hispanic or Latino ethnicity. By design, participants were drawn in near-equal numbers from the four U.S. Census regions. Compared with participants who completed all three waves (*n* = 442), participants who did not provide any data three times (i.e., dropped out after Wave 1 or 2; *n* = 579) tended to be younger with a mean difference (*M*_diff_) of 7.11 years (t = −6.70, *p* < 0.001), and experience less stress, anxiety, and depression at Wave 1 (*M*_diff_ = 1.35, t = −3.56, *p* < 0.001; *M*_diff_ = 1.25, t = −3.37, *p* < 0.001; *M*_diff_ = 1.40, t = −3.46, *p* < 0.001). No other comparisons between dropouts and completers were significant.

### 2.2. Measures

*Mental health outcome variables*. Mental health outcomes were measured using: (1) the Depression, Anxiety, and Stress Scale (DASS-21) [19], which was assessed at all three time points; and (2) the Posttraumatic Growth Inventory (PTGI) [20], which was assessed at Wave 3. The DASS-21 assesses symptoms of depression (e.g., dispirited, pessimistic, unable to experience joy), anxiety (e.g., apprehensive, shaky, pounding heart), and stress (e.g., tension, impatience, irritability), whereas the PGTI focuses on positive outcomes of coping with traumatic events (e.g., relating to others, new possibilities, personal strength, spiritual change, appreciation of life). Results of a confirmatory factor analysis (CFA) showed that a three-factor structure was a good fit to the data for the DASS-21: χ^2^ = 609.31, *df* = 181, CFI = 0.98, RMSEA = 0.048 (90% CI [0.044, 0.052], SRMR = 0.02 (Wave 1); χ^2^ = 498.97, *df* = 181, CFI = 0.97, RMSEA = 0.053 (90% CI [0.047, 0.058], SRMR = 0.02 (Wave 2); χ^2^ = 503.35, *df* = 181, CFI = 0.96, RMSEA = 0.063 (90% CI [0.057, 0.070], SRMR = 0.03 (Wave 3). For the PTGI, we fitted and compared a five-factor model (see [20]) and a second-order model. Fit indices were nearly identical for the two models. The second-order model, however, showed a lower BIC value compared to the five-factor model. We thus selected the second-order model as our final model: χ^2^ = 514.80, *df* = 184, CFI = 0.96, RMSEA = 0.064 (90% CI [.057, 0.070], SRMR = 0.03, and used PTGI as a unidimensional scale (i.e., computed as sum of all items). 

*Psychosocial predictors*. Except for perceived social strain, which was measured only at Wave 3, all other psychosocial predictors were measured at all three waves. Perceived health and financial risk were each measured with two 11-point Likert-scale items adapted from Griffin et al. [21]: “How likely is it that the COVID-19 pandemic will harm your health/financial security?” (0 = will certainly not harm my health/financial security and 10 = certain to harm my health/financial security) and “If you develop COVID-19/if the COVID-19 pandemic were harmful to your financial security, how serious do you think the harm would be?” (0 = not serious at all and 10 = as serious as it could possibly be). Each variable was modeled as the product of the two items which assessed perceived susceptibility and perceived severity respectively. Rumination was assessed using three 5-point Likert-scale items (e.g., “I can’t stop thinking about the COVID-19 pandemic;” 1 = strongly disagree, 5 = strongly agree). Co-rumination was also measured with three 5-point Likert scale items (e.g., “In any interaction I have, I am usually talking about the COVID-19 pandemic;” 1 = strongly disagree, 5 = strongly agree). Perceived social support was measured using the brief form of the Perceived Social Support Questionnaire (F-SozU K-6) [22] (e.g., “I receive a lot of understanding and security from others;” 1 = not true at all, 5 = very true). Perceived social strain was assessed by asking participants to first list the initials of up to seven people who typically provide them with support and then indicate how often in the past month each initial listed was a source of strain for them (e.g., making demands, criticizing you, letting you down, or getting on your nerves)” (1 = not at all, 5 = very often). The variable was computed by dividing the sum of perceived social strain for all listed initials by the number of initials listed.

*Behavioral predictors*. At each wave, participants were asked to indicate their adherence to national guidelines distributed nationally via postcard on March 16 (The President’s Coronavirus Guidelines for America, 2020) and the guideline issued by the CDC on April 3 on mask-wearing [1] in the past two weeks on a scale where 1 = not at all, 5 = completely with an additional “does not apply” option. Adherence was computed by averaging the score for all items where participants did not indicate “does not apply.” Coping strategy was measured using items (e.g., “Find activities to help me keep the event off my mind;” 1 = not at all, 7 = to a great extent) adapted from the Perceived Ability to Cope with Trauma (PACT) scale [23]. This scale assesses two general types of coping strategies: forward-focused, which has an optimistic focus on moving forward after the traumatic event, and trauma-focused, which focuses on processing the trauma. CFA results showed that three items had low factor loadings (i.e., <0.40). Dropping these items, the two-factor model reflecting the same structure as in [23] showed a good fit to the model: χ^2^ = 290.34, *df* = 110, CFI = 0.95, RMSEA = 0.061 (90% CI [0.052, 0.070], SRMR = 0.05. A composite score was created for forward-focused strategy and trauma-focused strategy respectively by averaging the corresponding items.

*Demographic predictors*. Political orientation was assessed by asking participants to rate themselves on a scale ranging from 1 = extremely liberal to 7 = extremely conservative. Details of the other demographic predictors are provided in Table 2. Employment status and COVID-19 symptoms and testing were measured at all waves. All other demographic predictors were measured once at Wave 1.

### 2.3. Statistical Analyses

Repeated measures ANOVAs were conducted to compare the mean scores of continuous predictors and outcomes across waves. Marginal homogeneity tests were used to examine the proportional change of categorical variables. Correlations were performed to assess relationships between variables across time points. A series of multiple linear regressions were conducted to assess the relationships between the psychosocial, behavioral, and demographic predictors and mental health outcomes. Standardized regression coefficients were reported. All analyses were performed in R [24], and *p* < 0.05 was employed as the threshold for statistical significance.

## 3. Results

### 3.1. Mental Health Outcomes over Time

Table 3 summarizes the descriptive statistics for each subscale of the DASS-21 at all three waves and the PTGI at Wave 3, significant differences across waves (as compared using repeated measures ANOVA), and the bivariate correlations between these outcomes. Depression, anxiety, and stress were highly correlated with each other across all waves. PTGI was correlated with stress and anxiety at all three waves, but not depression. Stress at Wave 3 was significantly lower than stress at Wave 1 and Wave 2. Depression at Wave 2 and Wave 3 were both significantly lower than depression at Wave 1. Similarly, anxiety at Wave 2 and Wave 3 were also both significantly lower than anxiety at Wave 1. No other comparisons were significant. Compared with norms developed from prior research with DASS scales (for which it is recommended to multiply the DASS-21 scores by two) [19], participants, on average, scored in the normal range on stress (full scale range: 0–21) in all three waves. At Wave 1, 56.92% of the participants reported normal stress levels, 8.62% had mild stress, 11.85% had moderate stress, 14.10% had severe stress, and 9.40% had extremely severe stress. At Wave 2, 59.72% of the participants were at normal stress levels, 9.32% had mild stress, 12.32% had moderate stress, 10.90% had severe stress, and 7.74% had extremely severe stress. At Wave 3, 64.48% of the participants were in the normal range, 6.56% had mild stress, 12.44% had moderate stress, 11.54% had severe stress, and 4.98% had extremely severe stress. Participants reported moderate depression (full scale range: 0-21) at Wave 1 (with 44.37% normal scores, 8.23% having mild depression, 15.38% having moderate depression, 11.46% having severe depression, and 20.57% having extremely severe depression), which decreased to a milder level at Waves 2 and 3. Specifically, at Wave 2, 50.08% of the participants were in the normal range, 8.69% had mild depression, 15.17% had moderate depression, 10.27% had severe depression, and 15.80% had extremely severe depression; at Wave 3, 55.88% had normal levels, 7.92% had mild depression, 11.54% had moderate depression, 9.28% had severe depression, and 15.38% had extremely severe depression Participants also reported, on average, a moderate level of anxiety (full scale range: 0–21) at Wave 1 (with 48.38% normal, 6.86% mild anxiety, 10.38% moderate anxiety, 8.03% severe anxiety, and 26.34% with extremely severe anxiety), which then also decreased to milder levels at Waves 2 and 3. Specifically, at Wave 2, 57.82% of the participants were in the normal range, 4.11% had mild anxiety, 9.16% had moderate anxiety, 5.85% had severe anxiety, and 23.06% had extremely severe anxiety; at Wave 3, 62.44% were in the normal range, 3.85% had mild anxiety, 9.05% had moderate anxiety, 3.39% had severe anxiety, and 21.27% had extremely severe anxiety.

### 3.2. Changes in Predictors over Time

Table 4 and Table 5 present the descriptive statistics and bivariate correlations between all psychosocial predictors and all behavioral predictors, respectively. A series of repeated measures ANOVA were conducted to examine mean differences in the predictors assessed at multiple waves; these statistics are reported in Table 4 and Table 5. A series of marginal homogeneity tests were performed to examine the proportional change in the demographic features assessed at multiple waves; these statistics are reported in Table 2. 

*Psychosocial predictors.* Perceived risk to health did not change significantly across waves. Perceived financial risk was significantly lower at Waves 2 and 3 compared to Wave 1. Rumination at Wave 2 was significantly lower compared to rumination at Wave 1 and rumination at Wave 3 was also significantly lower than at Wave 2. Similarly, co-rumination decreased significantly across waves. Perceived support was significantly higher at Wave 3 than Wave 1, but did not differ from Wave 2. Social strain did not change significantly across time.

*Behavioral predictors.* Adherence to national guidelines was significantly lower at Wave 3 compared to that at Waves 1 and 2. The difference between Wave 1 and Wave 2 was not significant. 

*Employment.* Results of the marginal homogeneity test suggested that there was a significant change in the proportion of participants’ employment status from Wave 1 to Waves 2 and 3. This significant global effect, however, could not be pinpointed to any specific categories as none of the post-hoc pairwise tests were significant. No significant change in proportion was observed from Wave 2 to 3 (see Table 2 for distribution across waves). 

*COVID-19 symptoms and testing.* Results of the marginal homogeneity test showed that there was no significant change in the proportion of participants’ experience of COVID-19-consistent symptoms (i.e., yes vs. no vs. not sure) across waves (see Table 2). The proportion of participants’ testing of COVID-19, however, changed significantly from Wave 1 to Waves 2 and 3. Post-hoc pairwise comparison showed that there was a significant change from having not been tested for COVID-19 at Wave 1 to having tested negative for COVID-19 at Waves 2 and 3. No other pairwise comparison was significant. The global change in COVID-19 testing status from Wave 2 to 3 was non-significant (see Table 2).

### 3.3. Demographic Predictors of Mental Health Outcomes

Table 2 and Table 6 present the standardized coefficients from two sets of regression analyses involving the demographic predictors (see also Supplementary Materials Tables S1 and S2 for confidence interval of the coefficients). The first set of coefficients were obtained by regressing each demographic predictor separately on the mental health outcomes at each wave, and thus represent “zero-order” effects of these variables (see Table 2). The second set of coefficients were obtained with “full-predictor” analyses: simultaneously regressing all of the predictors (demographic, psychosocial, behavioral) on the outcomes at each wave, and thus determining the effects of each demographic variable controlling for all other predictors (see Table 6). Both were included for their value in comparing findings across studies that report zero-order demographic effects, and for identifying the strongest demographic effects.

*Gender.* The zero-order models showed that, compared to males, females experienced less stress and depression at Wave 1 and less anxiety at all waves, along with less post-traumatic growth. However, in the full-predictor model, the only significant finding was that females experienced more stress than males at Wave 2.

*Age.* In both analyses, age negatively predicted stress, depression, and anxiety at all three time points.

*Race.* In the zero-order models, compared to White participants, Asian participants reported greater stress, depression, and posttraumatic growth at Wave 3 and greater anxiety at all three waves. Participants who self-identified as more than one race reported greater depression at Waves 2 and 3 compared to White participants. In addition, participants who self-identified as Black or African-American reported greater posttraumatic growth compared to those who self-identified as White. In the full-predictor models, compared to White participants, participants who self-identified as Black or African-American reported less stress at Waves 2 and 3 and greater posttraumatic growth.

*Ethnicity.* In the zero-order analyses, participants who self-identified as Hispanic or Latino experienced greater anxiety at Wave 1 and posttraumatic growth at Wave 3 compared to non-Hispanic or Latino participants. In the full-predictor models, participants who self-identified as Hispanic or Latino experienced less stress and anxiety at Wave 2 and greater posttraumatic growth at Wave 3 compared to non-Hispanic or Latino participants. 

*Education.* In the zero-order models, education was not a predictor of any outcomes, but in the full model, higher levels of education predicted greater anxiety at Wave 1. 

*Region*. In the zero-order models, compared to people residing in the northeast region, Midwest residents reported less depression at Wave 2, less stress at Waves 1 and 2, and less anxiety at all three time points. However, in the full-predictor models, region of residence did not predict any mental health outcomes.

*Political party and orientation*. In the zero-order models, at Wave 1, participants who self-identified as Democrat or Independent reported less depression, and Independents also reported less stress compared to those who strongly identified with the Republican Party. Democratic-leaning Independents and Republican-leaning Independents both reported less anxiety than strong Republicans. At Wave 2, Republican-leaning Independents again reported less anxiety than strong Republicans. Participants who were more conservative reported lower levels of depression at Wave 1 and lower levels of anxiety, depression, and stress at Wave 2. In the full-predictor models, there were fewer effects for political party or orientation. At Wave 2, participants who self-identified as Republicans reported greater stress and anxiety compared to Democrats. Participants who self-identified as Independents also reported greater stress at Wave 2 compared to Democrats. Participants who were more conservative also reported lower levels of stress and anxiety at Wave 2 and greater posttraumatic growth at Wave 3.

*Pre-existing health conditions.* In the zero-order analysis, individuals who had more pre-existing health conditions reported greater stress, anxiety, and depression at all three Waves. These effects all persisted in the full-predictor models.

*COVID-19 symptoms and testing.* In the zero-order models, compared to those who did not have any symptoms consistent with COVID-19, those who experienced COVID-19-consistent symptoms showed greater anxiety, depression, and stress at all waves. This group also reported greater posttraumatic growth. People who were unsure if they had symptoms consistent with COVID-19 also scored higher on all three DASS-21 subscales except stress at Wave 2. This group, however, reported less posttraumatic growth compared to people who did have any COVID-19-consistent symptoms. Different effects emerged in the full-predictor models. At Wave 1, participants who reported having tested negative for COVID-19 reported greater anxiety compared to those who had not tested for COVID-19. They also reported comparatively greater posttraumatic growth at Wave 3.

*Employment.* In the zero-order models, compared to people who were unemployed prior to the pandemic, those who reported being full-time employed by someone else scored higher on all mental health outcomes. People who reported being part-time employed by someone else also reported greater stress and anxiety at Wave 1. Those who were full-time employed reported higher levels of stress, depression, and anxiety at Wave 1 as well as higher levels of posttraumatic growth at Wave 3. People who were laid off, furloughed, or otherwise unemployed due to COVID-19 experienced greater stress at Wave 1. In the full-predictor models, two effects persisted: compared to people who were unemployed prior to the pandemic, those who reported being full-time employed by someone else or full-time self-employed scored higher on posttraumatic growth.

### 3.4. Psychosocial and Behavioral Predictors of Mental Health Outcomes

Table 7 presents the standardized coefficients for each psychosocial and behavioral predictor from the “full-predictor” analyses for each Wave (see also Supplementary Materials Table S3 for confidence interval of the coefficients). In these analyses, all predictors (demographic, psychosocial, behavioral) were simultaneously regressed on the outcomes, thus assessing the effects of each predictor controlling for all other predictors. For predictors assessed at multiple waves, regression analyses were conducted with wave-specific predictors (e.g., Wave 1 mental health outcomes were regressed on Wave 1 assessments of risk, rumination, social support, etc., whereas Wave 2 mental health outcomes were regressed on Wave 2 assessments).

Results showed that perceived health risk positively predicted anxiety at Waves 1 and 2. Perceived financial risk positively predicted stress at all three times and depression at Wave 3. Rumination positively predicted stress and depression at all waves. Co-rumination positively predicted stress at Wave 1 and stress, depression, and anxiety at Wave 2. Perceived social support negatively predicted depression at all times and stress at Wave 3. Perceived social strain positively predicted stress, depression, and anxiety at all three waves. Posttraumatic growth was positively predicted by perceived health risk, perceived social support, and perceived social strain. Adherence to national guidelines negatively predicted stress at all three times and anxiety at Wave 1. Use of the forward-focused coping strategy negatively predicted depression at all three waves and stress at Wave 2 and positively predicted posttraumatic growth. Use of trauma-focused coping strategy positively predicted depression at Time 1 and posttraumatic growth. 

## 4. Discussion

The current study examined the impact of COVID-19 and related events on mental health in the U.S. general public during April and May 2020. The average impact during this period appears to have been modest at the start, and trended downward over time. Symptoms of stress, anxiety, and depression were highest at Wave 1 (data collected April 20, or approximately one month into the US-based experience of the pandemic) and declined over the two remaining waves, with stress remaining in the “normal” range of the DASS throughout, and anxiety and depression falling from “moderate” to “mild” levels. Although these findings diverge somewhat from polls suggesting slightly higher levels of stress, anxiety, or depression during the survey period, this divergence is likely to reflect the distinction between the type and number of items used for public polling (e.g., “In the past seven days, have you felt anxiety, depression, loneliness or hopelessness?” [25]) and the DASS-21’s more conservative, symptom-based assessment. At the same time, despite averages in the normal range and the downward trends across waves, substantial percentages of our participants reported experiencing severe or extremely severe symptoms, particularly of depression and anxiety. These findings align with reports from other countries [14,15,16,17] and with the concerns of mental health professionals [2,3,4,5]; they also underscore the value of examining the predictors of mental distress during the pandemic.

### 4.1. Demographic Factors

The strongest, most consistent demographic effects on mental health came from pre-existing health conditions and age. The influence of pre-existing health conditions is unsurprising, given that COVID-19 was recognized very quickly as more likely to be severe or deadly for those with such conditions, and warnings to this effect were incorporated in the national public health guidelines. Despite the association between age and COVID-19 mortality, greater age was associated with less stress, depression, and anxiety, in both zero-order and full-predictor models. This finding is likely to reflect the relationship between age and emotional stability [26], along with ways in which the negative impact of COVID-19 was especially strong for younger people (e.g., colleges closed, part-time and “gig” work affected, lower likelihood of savings or health insurance to rely on, social and entertainment opportunities constricted; [27,28,29]). It is also consistent with research showing greater anxiety and depression in younger Chinese experiencing the pandemic [10]. 

Strikingly, race and ethnicity did not have strong or consistent effects on mental health, and the effects that emerged included greater post-traumatic growth for Black and Hispanic/Latino populations. However, the data collection predated both the eruption of Black Lives Matter protests following the death of George Floyd (May 25), and the emergence of data indicating that COVID-19 was disproportionately affecting communities of color [30]. From a present-day perspective, it is also striking that differences owing to political identification were relatively weak and inconsistent across outcomes, but a muted influence of political party aligns with data collection in April and May, prior to heightened politicization of the management and impact of reopening, and prior to the significant upturn in U.S. COVID-19 cases and deaths that occurred after an initial “flattening of the curve” [31]. It will be important that subsequent studies continue to examine how key demographic factors are associated with mental health as the pandemic’s impact continues to be felt.

### 4.2. Psychosocial and Behavioral Factors

Generalizing across the findings, it is clear that mental health outcomes for the general public in the U.S. were influenced by psychosocial and behavioral factors. Collectively, these findings are consistent with research on past pandemics and research from China and elsewhere on COVID-19, indicating that mental health during and after a pandemic is influenced by perceived risk, threat, and fear associated with the disease [32,33,34], rumination and co-rumination [35], social support [32], coping behaviors [36], and health-protective behaviors [12].

It is also the case that the various psychosocial and behavioral factors we assessed predicted different mental health outcomes in differing ways, despite strong correlations between stress, anxiety, and depression. For example, perceived health risk predicted anxiety at Waves 1 and 2, along with post-traumatic growth at Wave 3, but did not predict stress or depression, whereas perceived financial risk predicted stress but not anxiety or post-traumatic growth, and only predicted depression at Wave 3. These findings are interpretable with regard to the uncertainty associated with these types of risk—susceptibility to the health risk of COVID-19 is fraught with uncertainty, which generates anxiety about the future, whereas some type of financial risk or harm from COVID-19 is already a reality for many people, thus contributing to stress rather than anxiety. The minimal influence of risk perceptions on depression observed in this study differs from recently-released research with COVID-19 healthcare workers in Turkey [34], but this difference may be a function of lower risk perceptions (on average) in a general-public sample, or insufficient duration of perceived risk to generate depression; research on the SARS pandemic suggests that depression is related to risk perceptions over a longer span of time [32].

Ruminating about COVID-19 was associated with stress and depression across all three waves. These findings align with the negative effects of rumination in regard to other stressors, including natural disasters [37,38], and with recent research from Turkey showing that rumination about COVID-19 had a negative impact on well-being [33]. Co-rumination predicted stress at Waves 1 and 2, and anxiety and depression at Wave 2, but was no longer predictive of any mental health outcomes at Wave 3. This pattern of results has no clear explanation, but may be related to the average decline in co-rumination over time, or to changes in the content of conversations related to COVID-19 (e.g., conversations about the pandemic may have focused on more neutral or positive topics). Co-rumination deserves further research attention as the pandemic progresses, insofar as repetitive and non-productive discussion of shared stressors generally exacerbates mental health issues [37] but this phenomenon has been most frequently studied in child and young adult populations [39].

Capturing the extent to which one perceives being supported by others, perceived social support was consistently and negatively associated with depression (and with stress at Wave 3), and positively associated with post-traumatic growth. This observation is consistent with a huge volume of evidence that perceiving others as available to provide support and assistance buffers the negative health impacts of stressful events (including natural disasters [40]), and promotes viewing those events as positive in various ways [41]. Indeed, research with Chinese college students documents a positive influence of support on anxiety related to COVID-19 [8]. In counterpoint to the beneficial effects of social support, social strain (which included making demands, criticizing, letting down, and getting on one’s nerves) was the strongest and most consistent predictor of health outcomes across waves, correlated with heightened stress, anxiety, and depression, and with post-traumatic growth (albeit more weakly). The negative impact of social strain on mental health is well-documented in other contexts [42], but deserves continued focus in the wake of COVID-19, given the probability that strain is heightened under stay-at-home orders that simultaneously create unwanted togetherness with some (i.e., co-residents) and distance from others.

Forward-focused coping, including behaviors such as looking for a silver lining, distracting attention from the event and its associated emotions, and following a regular schedule and engaging in enjoyable activities, was protective against depression at all waves (and stress at Wave 2), whereas trauma-focused coping was positively associated with depression at Wave 1. Both types of coping were positively associated with post-traumatic growth at Wave 3. The demonstrated value of forward-focused coping aligns with research on people quarantined in Spain [36], showing efforts to sustain a healthy lifestyle (e.g., balanced diet, maintaining routines, time outdoors, avoiding COVID-19 news) were protective against anxiety and depression. The inconsistent effect of trauma-focused coping is not unexpected given the evidence that the effect of trauma-focused coping changes over time [43]. In the context of COVID-19, it is possible that effortful processing of the pandemic at the initial stages where it was characterized with high novelty and uncertainty was particularly overwhelming and thus depression-inducing. Over time, however, as it became increasingly normal to cognitively process the trauma, the negative effect might have begun to fade.

Adherence to national guidelines predicted reduced stress across all three waves, and anxiety at Wave 1. This finding is consistent with research indicating that Chinese individuals who adopted precautionary measures such as hand-washing experienced less stress, anxiety, and depression [12]. More broadly, it indicates that taking principled action to protect physical health from risk is also beneficial to mental health.

## 5. Limitations

This study has several limitations. Budget constraints motivated our use of Qualtrics.com, which provides national U.S. samples that tend to be representative demographically and politically, but are not random [44]. Our findings may thus reflect biases in our sample. In addition, our sample was diminished by attrition across waves, especially between Wave 1 and Wave 2, and we were unable to measure all constructs at all waves. The data were collected during April and May of 2020, but we do not know if the relationships observed will be stable as the pandemic proceeds and new developments occur, including a rising death toll in the U.S. Lastly, as the data did not contain any pre-pandemic baseline measures, we were not able to establish any causal relationship between the COVID-19 pandemic and the mental health outcomes. 

## 6. Conclusions

Limitations notwithstanding, our findings suggest that the early months of the U.S.’ experience of the pandemic were characterized by a modest mental health impact in the general public, an effect that was more pronounced for younger people and those with pre-existing health conditions, and for those experiencing greater perceived risk, higher levels of rumination or co-rumination, less social support, and greater social strain. More positive mental health was associated with the adoption of coping strategies, especially those that were forward-looking, and with greater adherence to national health-protection guidelines. 

It is important to note that our Wave 3 data collection coincided with the leveling-off or decline in reported COVID-19 cases and deaths in many states in mid-May [45], and with states “reopening,” or announcing plans for reopening [46]. As such, lowering levels of mental distress may help explain increasingly less cautious behavior exhibited by the American public between mid-May and the present [47] insofar as people who are less stressed or anxious about COVID-19 are likely to undertake fewer health-protective measures [48]. Unfortunately, with early efforts to “flatten the curve” largely abandoned and exponential growth occurring in many states (as of August, 2020), it seems probable that the US was both insufficiently stressed in March and April to promote long-term health-protective attitudes, and that there will be as-yet-determined mental health effects associated with COVID-19 illness, death, job loss, and renewed efforts to contain the spread. 

As the pandemic continues, our study suggests that public health interventions can be focused on heightening perceived health risk [49], promoting adherence to health-protective guidelines such as mask-wearing [50], discouraging non-productive rumination and co-rumination [51], encouraging social support and reducing social strain [52], and guiding on healthy coping strategies that focus on the future [53]. This will be no easy task, and one that will require coordinated, sustained efforts from all areas of society. 

## Figures and Tables

**Table 1:** Sample demographic statistics (*N*_wave1_ = 1021).

Demographic Characteristics	Mean	SD	*n*	Percentage (%)
Age (years)	45.30	16.46		
18–29			202	19.78
30–39			233	22.82
40–49			172	16.85
50–59			157	15.38
60–69			176	17.24
70 or older			81	7.93
Sex				
Female			534	52.30
Male			483	47.31
Non-binary			4	0.39
Race				
White/Caucasian			764	75.05
Black or African American			109	10.71
Asian			66	6.48
More than one race			24	2.36
American Indian or Alaska Native			17	1.67
Native Hawaiian or other Pacific Islander			3	0.29
other or prefer not to answer			35	3.44
Ethnicity				
Hispanic/Latino			99	9.76
Census Region				
Midwest			250	24.49
Northeast			252	24.68
South			261	25.56
West			258	25.27

**Table 2:** Simple Regression of Mental Health Outcomes on Demographic Predictors and Change in Demographic Predictors over Time.

	Wave 1 (*N* = 1021)			Wave 2 (*N* = 633)			Wave 3 (*N* = 442)			
	Stress	Depression	Anxiety	Stress	Depression	Anxiety	Stress	Depression	Anxiety	PTG
*Gender*										
Female	−0.12 ***	−0.10 **	−0.15 ***	−0.04	−0.02	−0.12 **	−0.05	−0.05	−0.1 2 *	−0.10 *
Male	Ref	Ref	Ref	Ref	Ref	Ref	Ref	Ref	Ref	Ref
*Age*	−0.32 ***	−0.33 ***	−0.33 ***	−0.33 ***	−0.33 **	−0.38 ***	−0.34 ***	−0.33 ***	−0.38 ***	−0.08
*Race*										
American Indian or Alaska Native	−0.01	−0.004	−0.02	−0.001	−0.02	0.04	0.001	−0.01	−0.004	0.03
Asian	0.03	0.03	0.06 *	0.05	0.06	0.10 *	0.13 **	0.12 **	0.15 **	0.11 *
Native Hawaiian or other Pacific Islander	0.06	0.03	0.06	−−	−−	−−	−−	−−	−−	−−
Black or African American	−0.05	−0.02	−0.01	−0.02	−0.003	0.03	−0.03	−0.01	0.02	0.16 ***
More than one race	0.03	0.03	0.01	0.05	0.10 *	0.06	0.08	0.11 *	0.08	−0.03
Other	0.02	0.02	0.02	0.002	−0.003	0.03	−0.004	0.01	0.03	0.08
White	Ref	Ref	Ref	Ref	Ref	Ref	Ref	Ref	Ref	Ref
*Ethnicity*										
Hispanic/Latino	0.04	0.06	0.08 *	0.004	0.04	0.02	0.01	0.05	0.05	0.10 *
Not Hispanic/Latino	Ref	Ref	Ref	Ref	Ref	Ref	Ref	Ref	Ref	Ref
*Education*	0.04	0.02	0.07 *	−0.03	−0.04	0.01	0.04	−0.004	0.03	−0.04
*Region of residence*										
Midwest	−0.09 *	−0.07	−0.10 *	−0.11 *	−0.11 *	−0.12 *	−0.08	−0.09	−0.12 *	−0.08
South	−0.06	−0.05	−0.07	−0.06	−0.06	−0.04	−0.07	−0.07	−0.01	0.05
West	−0.02	0.02	−0.02	−0.07	−0.05	−0.07	−0.05	−0.02	−0.05	−0.10
Northeast	Ref	Ref	Ref	Ref	Ref	Ref	Ref	Ref	Ref	Ref
*Party identification*										
Republican	0.06	0.05	0.05	0.03	0.01	0.01	−0.05	0.02	0.01	−0.03
Independents	−0.02	−0.02	−0.04	−0.04	−0.04	−0.09 *	−0.04	−0.001	−0.08	−0.14 **
Third party	−0.02	−0.02	−0.04	−0.01	0.01	−0.02	−0.05	−0.04	−0.04	−0.03
*Democratic*	Ref	Ref	Ref	Ref	Ref	Ref	Ref	Ref	Ref	Ref
*Political orientation*	−0.05	−0.07 *	−0.05	−0.09 *	−0.09 *	−0.09 *	−0.03	−0.05	−0.07	0.07
*Health condition*	0.14 ***	0.14 ***	0.18 ***	0.22 ***	0.21 ***	0.24 ***	0.17 ***	0.21 ***	0.22 ***	0.07
*COVID-19 symptom*										
Yes	*n* = 89, 8.75%			*n* = 57, 9.02%			*n* = 28, 6.33%			
Yes	0.14 ***	0.13 ***	0.16 ***	0.15 ***	0.16 ***	0.19 ***	0.12 **	0.13 **	0.15 **	0.10 *
Unsure	*n* = 30, 2.95%			*n* = 19, 3.01%			*n* = 14, 3.17%			
Unsure	0.10 **	0.09 **	0.10 **	0.07	0.08 *	0.12 **	0.11 *	0.11 *	0.11 *	−0.10 *
No	*n* = 898, 88.30%			*n* = 556, 87.97%			*n* = 400, 90.50%			
No	Ref	Ref	Ref	Ref	Ref	Ref	Ref	Ref	Ref	Ref
*COVID-19 testing*										
Yes, negative	*n* = 55, 5.41% ^a^			*n* = 40, 6.33% ^b^			*n* = 27, 6.13% ^b^			
Yes, negative	0.19 ***	0.18 ***	0.22 ***	0.13 ***	0.12 **	0.20 ***	0.13 **	0.11 *	0.17 ***	0.13 **
Yes, positive	*n* = 11, 1.08%			*n* = 7, 1.11%			*n* = 6, 1.36%			
Yes, positive	0.10 ***	0.10 **	0.15 ***	0.08 *	0.09 *	0.12 **	0.14 **	0.12 **	0.18 ***	0.10 *
Yes, waiting	*n* = 7, 0.69%			*n* = 6, 0.95%			*n* = 3, 0.68%			
Yes, waiting	0.07 *	0.07 *	0.08 **	0.09 *	0.08 *	0.12 **	0.05	0.04	0.07	0.04
No	*n* = 944, 92.82% ^a^			*n* = 579, 91.61% ^b^			*n* = 404, 91.82% ^b^			
No	Ref	Ref	Ref	Ref	Ref	Ref	Ref	Ref	Ref	Ref
*Employment status*										
Full-time	*n* = 302, 30.32%			*n* = 220, 34.98%			*n* = 156, 35.37%			
Full-time employed by someone else	0.11 **	0.08 *	0.12 **	0.13 **	0.10 *	0.20 ***	0.18 ***	0.14 **	0.21 ***	0.12 *
Part-time	*n* = 87, 8.73%			*n* = 50, 7.95%			*n* = 27, 6.12%			
Part-time employed by someone else	0.10 **	0.03	0.08 *	−0.01	−0.06	0.01	0.004	−0.05	−0.04	0.09
Self-employed	*n* = 174, 17.47%			*n* = 78, 12.40%			*n* = 54, 12.24%			
Full-time self-employed	0.11 **	0.09 *	0.08 *	0.07	0.06	0.07	0.09	0.05	0.04	0.14 **
Unemployed-C-19	*n* = 103, 10.34%			*n* = 45, 7.15%			*n* = 37, 8.39%			
Laid off, furloughed, or otherwise unemployed due to COVID-19	0.08 *	0.04	0.07	0.03	−0.003	0.04	0.09	0.04	0.06	0.10
Unemployed prior	*n* = 330, 33.13%			*n* = 236, 37.52%			*n* = 167, 37.87%			
Unemployed prior to COVID-19	Ref	Ref	Ref	Ref	Ref	Ref	Ref	Ref	Ref	Ref

*Note*. PTG = Posttraumatic growth. All coefficients are standardized. *** *p* < 0.001, ** *p* < 0.01, * *p* < 0.05. Ref = Reference group. Descriptive statistics with differing superscripts (i.e., ^a^ vs. ^b^) changed significantly in proportion, *p* < 0.05.

**Table 3:** Means, Standard Deviations, and Bivariate Correlations of Mental Health Outcomes.

	*M*	*SD*	1	2	3	4	5	6	7	8	9
1. StressT1	7.39 ^a^	6.03	--								
2. StressT2	6.77 ^a^	5.83	0.70 ***	--							
3. StressT3	6.13 ^b^	5.60	0.75 ***	0.74 ***	--						
4. DepressionT1	7.14 ^a^	6.48	0.87 ***	0.68 ***	0.71 ***	--					
5. DepressionT2	6.26 ^b^	6.13	0.65 ***	0.85 ***	0.69 ***	0.76 ***	--				
6. DepressionT3	5.69 ^b^	6.06	0.68 ***	0.66 ***	0.88 ***	0.77 ***	0.76 ***	--			
7. AnxietyT1	5.77 ^a^	5.95	0.85 ***	0.63 ***	0.66 ***	0.83 ***	0.62 ***	0.65 ***	--		
8. AnxietyT2	4.90 ^b^	5.64	0.68 ***	0.86 ***	0.67 ***	0.68 ***	0.82 ***	0.65 ***	0.73 ***	--	
9. AnxietyT3	4.36 ^b^	5.42	0.67 ***	0.67 ***	0.86 ***	0.68 ***	0.66 ***	0.84 ***	0.74 ***	0.75 ***	--
10. PTG	58.34	26.76	0.16 ***	0.10 *	0.11 *	0.06	0.03	0.03	0.24 ***	0.21 ***	0.18 ***

*Note*. PTG = Posttraumatic growth. *** *p* < 0.001, * *p* < 0.05. Stress, depression, and anxiety were Depression, Anxiety and Stress (DASS-21) subscales. Means with differing superscripts (i.e., ^a^ vs. ^b^) within each DASS-21 subscale are significantly different from each other, *p* < 0.05.

**Table 4:** Means, Standard Deviations, and Bivariate Correlations of Psychosocial Predictors.

	M	SD	1	2	3	4	5	6	7	8	9	10	11	12	13	14	15	16
1.HRT1	46.21	28.82	NA															
2.HRT2	45.89	28.19	0.67 ***	NA														
3.HRT3	44.84	29.20	0.67 ***	0.68 ***	NA													
4.FRT1	52.75 ^a^	30.81	0.40 ***	0.24 ***	0.28 ***	NA												
5.FRT2	50.13 ^b^	30.85	0.34 ***	0.39 ***	0.35 ***	0.70 ***	NA											
6.FRT3	49.04 ^b^	30.00	0.33 ***	0.35 ***	0.45 ***	0.70 ***	0.78 ***	NA										
7.RMT1	3.66 ^a^	0.98	0.40 ***	0.37 ***	0.34 ***	0.33 ***	0.35 ***	0.33 ***	0.79									
8.RMT2	3.57 ^b^	1.04	0.39 ***	0.40 ***	0.35 ***	0.31 ***	0.37 ***	0.34 ***	0.76 ***	0.82								
9.RMT3	3.43 ^c^	1.05	0.37 ***	0.34 ***	0.40 ***	0.30 ***	0.38 ***	0.35 ***	0.71 ***	0.74 ***	0.81							
10.CRMT1	3.49 ^a^	1.04	0.35 ***	0.33 ***	0.34 ***	0.31 ***	0.35 ***	0.39 ***	0.73 ***	0.63 ***	0.66 ***	0.87						
11.CRMT2	3.38 ^b^	1.11	0.35 ***	0.36 ***	0.35 ***	0.34 ***	0.39 ***	0.42 ***	0.64 ***	0.75 ***	0.69 ***	0.73 ***	0.90					
12.CRMT3	3.27 ^c^	1.16	0.29 ***	0.31 ***	0.36 ***	0.32 ***	0.37 ***	0.39 ***	0.61 ***	0.63 ***	0.77 ***	0.74 ***	0.77 ***	0.90				
13.PST1	3.84 ^a^	0.98	0.05	0.08 *	−0.01	0.05	0.04	0.00	0.05	−0.02	−0.07	0.08 **	0.05	0.06	0.89			
14.PST2	3.89	0.92	0.02	0.02	−0.02	0.02	−0.01	−0.03	0.02	0.01	−0.03	0.06	0.09 *	0.07	0.64 ***	0.88		
15.PST3	3.94 ^b^	0.95	−0.04	−0.04	−0.08	0.01	−0.05	−0.04	−0.07	−0.09	−0.07	−0.03	−0.01	0.04	0.70 ***	0.73 ***	0.90	
16.Strain	2.06	1.16	0.11 *	0.15 **	0.19 ***	0.22 ***	0.25 ***	0.29 ***	0.17 ***	0.18 ***	0.16 ***	0.23 ***	0.23 ***	0.21 ***	−0.09	−0.16 **	−0.19 ***	NA

*Note.* HR = Perceived health risk; FR = Perceived financial risk; RM = Rumination; CRM = Co-rumination; PS = Perceived social support; Strain = Perceived social strain. *** *p* < 0.001, ** *p* < 0.01, * *p* < 0.05. Means with differing superscripts (i.e., ^a^ vs. ^b^ vs. ^c^) within each variable are significantly different from each other, *p* < 0.05. Reliability of each scale (italicized) is presented on the diagonal (NA = not applicable).

**Table 5:** Bivariate Correlations among Behavioral Predictors.

	M	SD	1	2	3	4	5
1.Adherence to national guidelines T1	4.36 ^a^	0.77	NA				
2.Adherence to national guidelines T2	4.42 ^a^	0.77	0.62 ***	NA			
3.Adherence to national guidelines T3	4.27 ^b^	0.76	0.63 ***	0.70 ***	NA		
4.Forward-focused coping strategy	4.97	1.10	0.30 ***	0.26 ***	0.33 ***	*0.91*	
5.Trauma-focused coping strategy	4.49	1.26	0.29 ***	0.26 ***	0.29 ***	0.70 ***	*0.84*

*Note.* *** *p* < 0.001. Means with differing superscripts (i.e., ^a^ vs. ^b^) for the variable “adherence to national guidelines” are significantly different from each other, *p* < 0.05. Coping strategies were assessed at T3 only. Reliability of each scale (italicized) is presented on the diagonal (NA = not applicable).

**Table 6:** Multiple Regression of Mental Health Outcomes on Demographic Predictors.

	Wave 1 (*N* = 1021)			Wave 2 (*N* = 633)			Wave 3 (*N* = 442)			
	Stress	Depression	Anxiety	Stress	Depression	Anxiety	Stress	Depression	Anxiety	PTG
*Gender*										
Female	0.07	0.05	0.04	0.06	0.05	−0.01	0.10 *	0.05	−0.0003	−0.02
Male	Ref	Ref	Ref	Ref	Ref	Ref	Ref	Ref	Ref	Ref
*Age*	−0.25 ***	−0.23 ***	−0.28 ***	−0.27 ***	−0.27 ***	−0.29 ***	−0.20 ***	−0.18 ***	−0.23 ***	0.01
*Race*										
American Indian or Alaska Native	−0.05	−0.04	−0.04	−0.06	−0.06	−0.01	−0.01	−0.03	−0.02	0.05
Asian	0.004	−0.02	0.03	−0.08	−0.05	−0.01	0.004	0.01	0.04	0.05
Native Hawaiian or other Pacific Islander	--	--	--	--	--	--	--	--	--	--
Black or African American	−0.07	−0.03	−0.07	−0.08 *	−0.05	−0.03	−0.12 **	−0.08	−0.08	0.09 *
More than one race	0.02	0.01	−0.03	−0.01	0.04	0.01	0.03	0.05	−0.0003	−0.01
Other	0.03	−0.03	0.01	0.02	−0.01	0.06	−0.03	−0.03	−0.02	0.003
White	Ref	Ref	Ref	Ref	Ref	Ref	Ref	Ref	Ref	Ref
*Ethnicity*										
Hispanic/Latino	−0.05	0.01	0.02	−0.11 *	−0.05	−0.11 *	−0.07	−0.02	−0.02	0.13 **
Not Hispanic/Latino	Ref	Ref	Ref	Ref	Ref	Ref	Ref	Ref	Ref	Ref
*Education*	0.01	−0.04	−0.02	−0.02	−0.02	−0.03	0.07	0.01	0.02	−0.06
*Region of residence*										
Midwest	0.02	0.01	0.03	−0.07	−0.07	−0.05	−0.02	−0.03	0.001	−0.02
South	−0.003	−0.03	0.02	−0.06	−0.08	−0.02	−0.06	−0.04	0.03	0.02
West	0.03	0.08	0.06	−0.02	−0.01	0.001	−0.03	0.004	−0.01	−0.07
Northeast	Ref	Ref	Ref	Ref	Ref	Ref	Ref	Ref	Ref	Ref
*Party identification*										
Republican	0.06	0.03	0.05	0.14 **	0.07	0.11 *	0.05	0.02	0.06	0.01
Independents	0.01	−0.01	−0.01	0.08 *	0.05	0.02	0.002	0.04	0.004	−0.06
Third party	0.01	0.001	0.02	0.02	0.01	0.04	0.002	0.001	0.03	0.03
*Democratic*	Ref	Ref	Ref	Ref	Ref	Ref	Ref	Ref	Ref	Ref
*Political orientation*	−0.003	0.01	0.002	−0.10 *	−0.04	−0.09 *	−0.03	−0.02	−0.07	0.12 **
*Health condition*	0.15 ***	0.20 ***	0.19 ***	0.14 ***	0.12 **	0.16 ***	0.13 **	0.19 ***	0.18 ***	0.01
*COVID-19 symptom*										
Yes	0.03	0.03	0.01	0.01	0.02	0.07	0.01	0.03	0.03	0.03
Unsure	−0.002	−0.01	−0.03	−0.01	0.01	0.05	0.02	0.01	0.04	0.01
No	Ref	Ref	Ref	Ref	Ref	Ref	Ref	Ref	Ref	Ref
*COVID-19 testing*										
Yes, negative	0.08	0.05	0.10 *	0.02	−0.01	0.03	0.004	−0.002	0.03	0.07 *
Yes, positive	0.03	0.01	0.07	0.02	0.02	0.03	0.03	0.01	0.06	0.02
Yes, waiting	0.03	0.03	0.03	0.03	0.01	0.04	−0.01	0.01	0.02	−0.03
No	Ref	Ref	Ref	Ref	Ref	Ref	Ref	Ref	Ref	Ref
*Employment status*										
Full-time employed by someone else	−0.01	0.01	0.001	−0.05	−0.05	−0.03	−0.05	−0.06	−0.05	0.09 *
Part-time employed by someone else	0.03	0.03	0.01	0.03	−0.01	−0.03	0.03	−0.02	−0.03	0.07
Full-time self-employed	0.07	0.07	0.03	0.06	0.02	0.001	0.04	0.03	0.004	0.11 **
Laid off, furloughed, or otherwise unemployed due to COVID-19	0.06	0.03	0.03	−0.01	−0.02	0.004	0.01	−0.02	0.004	0.07
Unemployed prior to COVID-19	Ref	Ref	Ref	Ref	Ref	Ref	Ref	Ref	Ref	Ref

*Note*. PTG = Posttraumatic growth. All coefficients are standardized. *** *p* < 0.001, ** *p* < 0.01, * *p* < 0.05. Ref = Reference group.

**Table 7:** Regression of Mental Health Outcomes on Psychosocial and Behavioral Predictors.

	Wave 1			Wave 2			Wave 3			
	Stress	Depression	Anxiety	Stress	Depression	Anxiety	Stress	Depression	Anxiety	PTG
Perceived health risk	0.08	0.01	0.12 *	0.04	0.08	0.10 *	0.001	−0.01	0.05	0.11 **
Perceived financial risk	0.11 *	0.08	0.03	0.10 *	0.07	0.06	0.13 **	0.10 *	0.05	−0.07
Rumination	0.17 **	0.19 **	0.10	0.16 **	0.13 *	0.08	0.23 ***	0.15 *	0.12	0.04
Co-rumination	0.14 *	0.11	0.12	0.25 ***	0.20 **	0.19 **	0.07	0.10	0.07	−0.01
Perceived social support	−0.08	−0.10 *	−0.02	−0.05	−0.10 *	−0.01	−0.12 **	−0.13 **	−0.03	0.16 ***
Perceived social strain	0.23 ***	0.22 ***	0.28 ***	0.29 ***	0.27 ***	0.33 ***	0.35 ***	0.34 ***	0.39 ***	0.08 *
Adherence to national guidelines	−0.10 *	−0.04	−0.10 *	−0.12 **	−0.07	−0.06	−0.12 **	−0.06	−0.08	0.01
Forward-focused coping strategy	−0.12	−0.26 ***	−0.08	−0.12 *	−0.20 ***	−0.05	−0.05	−0.20 ***	−0.07	0.43 ***
Trauma-focused coping strategy	0.11	0.15 *	0.12	0.04	0.06	−0.001	0.06	0.10	0.07	0.20 ***

*Note*. PTG = Posttraumatic growth. All coefficients are standardized. *** *p* < 0.001, ** *p* < 0.01, * *p* < 0.05.

## Supplementary Materials

The confidence interval for all standardized coefficients from the regression models is available as Supplementary Materials. The following are available online at https://www.mdpi.com/1660-4601/17/17/6315/s1, Table S1: 95% Confidence Interval for Standardized Coefficients from Simple Regression of Mental Health Outcomes on Demographic Predictors, Table S2: 95% Confidence Interval for Standardized Coefficients from Multiple Regression of Mental Health Outcomes on Demographic Predictors, Table S3: 95% Confidence Interval for Standardized Coefficients from Regression of Mental Health Outcomes on Psychosocial and Behavioral Predictors.
