# Supplementary material for: Mental Health and Its Predictors during the Early Months of the COVID-19 Pandemic Experience in the United States
Source: Int J Environ Res Public Health. 2020 Aug 31;17(17):6315. doi: 10.3390/ijerph17176315 (PMC7503583; doi:10.3390/ijerph17176315)
Supplement: Supplementary file 1 [file ijerph-17-06315-s001.pdf]

Table S1. 95% Confidence Interval for Standardized Coefficients from Simple Regression of Mental Health Outcomes on Demographic Predictors

	Wave 1 (N = 1021)			Wave 2 (N = 633)			Wave 3 (N = 442)			
	**Stress**	**Depression**	**Anxiety**	**Stress**	**Depression**	**Anxiety**	**Stress**	**Depression**	**Anxiety**	**PTG**
Gender										
Female	[-.18, -.06]	[-.16, -.03]	[-.21, -.09]	[-.11, .04]	[-.10, .06]	[-.20, -.04]	[-.15, .04]	[-.15, .04]	[-.22, -.03]	[-.20, -.01]
Male	Ref	Ref	Ref	Ref	Ref	Ref	Ref	Ref	Ref	Ref
Age	[-.37, -.26]	[-.39, -.27]	[-.39, -.27]	[-.40, -.25]	[-.41, -.26]	[-.45, -.30]	[-.42, -.25]	[-.42, -.24]	[-.47, -.30]	[-.17, .01]
Race										
American Indian or Alaska Native	[-.07, .05]	[-.07, .06]	[-.08, .04]	[-.08, .08]	[-.09, .06]	[-.04, .12]	[-.09, .09]	[-.11, .08]	[-.10, .09]	[-.06, .12]
Asian	[-.03, .09]	[-.03, .09]	[.00, .12]	[-.02, .13]	[-.02, .14]	[.02, .18]	[.04, .22]	[.03, .22]	[.06, .25]	[.02, .20]
Native Hawaiian or other Pacific Islander	[-.01, .12]	[-.03, .09]	[.00, .12]	--	--	--	--	--	--	--
Black or African American	[-.11, .02]	[-.09, .04]	[-.08, .05]	[-.10, .06]	[-.08, .08]	[-.05, .11]	[-.13, .06]	[-.10, .08]	[-.07, .12]	[.07, .26]
More than one race	[-.03, .10]	[-.03, .09]	[-.05, .07]	[-.03, .13]	[.02, .18]	[-.02, .13]	[-.01, .18]	[.01, .20]	[-.02, .17]	[-.12, .06]
Other	[-.04, .08]	[-.05, .08]	[-.04, .08]	[-.08, .08]	[-.08, .07]	[-.05, .11]	[-.10, .09]	[-.08, .11]	[-.06, .12]	[-.01, .17]
White	Ref	Ref	Ref	Ref	Ref	Ref	Ref	Ref	Ref	Ref
Ethnicity										
Hispanic/Latino	[-.02, .10]	[-.01, .12]	[.02, .14]	[-.07, .08]	[-.04, .11]	[-.05, .10]	[-.08, .11]	[-.04, .15]	[-.04, .14]	[.01, .20]
Not Hispanic/Latino	Ref	Ref	Ref	Ref	Ref	Ref	Ref	Ref	Ref	Ref
Education	[-.02, .10]	[-.05, .08]	[.01, .13]	[-.10, .05]	[-.12, .04]	[-.07, .09]	[-.05, .13]	[-.10, .09]	[-.06, .12]	[-.13, .05]
Region of residence										
Midwest	[-.16, -.01]	[-.14, .01]	[-.17, -.02]	[-.20, -.01]	[-.20, -.01]	[-.22, -.03]	[-.20, .03]	[-.21, .02]	[-.23, .00]	[-.20, .03]
South	[-.13, .02]	[-.12, .03]	[-.14, .01]	[-.15, .04]	[-.16, .03]	[-.13, .06]	[-.19, .04]	[-.19, .04]	[-.13, .10]	[-.07, .16]
West	[-.10, .05]	[-.05, .10]	[-.09, .06]	[-.17, .02]	[-.14, .05]	[-.16, .03]	[-.16, .07]	[-.13, .10]	[-.17, .06]	[-.22, .01]
Northeast	Ref	Ref	Ref	Ref	Ref	Ref	Ref	Ref	Ref	Ref
Party identification										
Republican	[-.01, .13]	[-.02, .12]	[-.02, .11]	[-.05, .12]	[-.08, .09]	[-.07, .09]	[-.05, .15]	[-.09, .12]	[-.09, .11]	[-.13, .07]
Independents	[-.09, .04]	[-.09, .04]	[-.11, .02]	[-.12, .04]	[-.13, .04]	[-.18, -.01]	[-.15, .06]	[-.10, .10]	[-.18, .02]	[-.24, -.04]
Third party	[-.08, .04]	[-.08, .05]	[-.10, .02]	[-.09, .07]	[-.06, .09]	[-.10, .06]	[-.15, .04]	[-.14, .05]	[-.13, .05]	[-.12, .06]
Democratic	Ref	Ref	Ref	Ref	Ref	Ref	Ref	Ref	Ref	Ref
Political orientation	[-.11, .02]	[-.13, .00]	[-.11, .01]	[-.17, -.01]	[-.17, -.01]	[-.17, -.01]	[-.13, .06]	[-.14, .05]	[-.17, .02]	[-.03, .16]
Health condition	[.08, .20]	[.08, .20]	[.12, .24]	[.14, .30]	[.13, .28]	[.17, .32]	[.08, .27]	[.12, .30]	[.13, .32]	[-.03, .16]
COVID-19 symptom										
Yes	[.08, .20]	[.07, .20]	[.10, .22]	[.07, .23]	[.08, .24]	[.11, .26]	[.03, .22]	[.04, .22]	[.06, .24]	[.00, .19]
Unsure	[.04, .16]	[.03, .15]	[.04, .16]	[.00, .15]	[.00, .16]	[.04, .19]	[.01, .20]	[.02, .20]	[.02, .20]	[-.20, -.01]
No	Ref	Ref	Ref	Ref	Ref	Ref	Ref	Ref	Ref	Ref
COVID-19 testing										
Yes, negative	[.13, .25]	[.12, .24]	[.16, .28]	[.06, .21]	[.04, .20]	[.13, .28]	[.04, .22]	[.02, .21]	[.07, .26]	[.04, .22]
Yes, positive	[.04, .16]	[.04, .16]	[.09, .21]	[.00, .16]	[.01, .17]	[.04, .19]	[.05, .23]	[.03, .22]	[.08, .27]	[.01, .19]
Yes, waiting	[.01, .13]	[.01, .13]	[.02, .14]	[.01, .17]	[.00, .16]	[.04, .19]	[-.05, .14]	[-.05, .13]	[-.03, .16]	[-.06, .13]
No	Ref	Ref	Ref	Ref	Ref	Ref	Ref	Ref	Ref	Ref
Employment status										

Full-time employed by someone else	[.04, .19]	[.01, .15]	[.05, .19]	[.04, .22]	[.02, .19]	[.11, .29]	[.08, .28]	[.03, .24]	[.11, .32]	[.01, .22]
Part-time employed by someone else	[.03, .16]	[-.04, .10]	[.01, .14]	[-.09, .08]	[-.14, .02]	[-.08, .09]	[-.09, .10]	[-.15, .04]	[-.14, .06]	[-.01, .19]
Full-time self-employed	[.04, .18]	[.02, .15]	[.01, .15]	[-.01, .15]	[-.03, .14]	[-.02, .15]	[-.01, .19]	[-.05, .15]	[-.06, .14]	[.04, .24]
Laid off, furloughed, or otherwise unemployed due to COVID-19	[.01, .14]	[-.03, .11]	[.00, .13]	[-.05, .11]	[-.09, .08]	[-.05, .12]	[-.01, .18]	[-.06, .14]	[-.03, .16]	[.00, .19]
Unemployed prior to COVID-19	Ref	Ref	Ref	Ref	Ref	Ref	Ref	Ref	Ref	Ref

Note. PTG = Posttraumatic growth. Ref = Reference group.

Table S2. 95% Confidence Interval for Standardized Coefficients from Multiple Regression of Mental Health Outcomes on Demographic Predictors

	Wave 1 (N = 1021)			Wave 2 (N = 633)			Wave 3 (N = 442)			
	**Stress**	**Depression**	**Anxiety**	**Stress**	**Depression**	**Anxiety**	**Stress**	**Depression**	**Anxiety**	**PTG**
Gender										
Female	[-.01, .15]	[-.04, .13]	[-.04, .12]	[-.02, .14]	[-.03, .13]	[-.09, .07]	[.01, .18]	[-.03, .13]	[-.08, .08]	[-.09, .06]
Male	Ref	Ref	Ref	Ref	Ref	Ref	Ref	Ref	Ref	Ref
Age	[-.35, -.14]	[-.33, -.13]	[-.38, -.18]	[-.37, -.18]	[-.37, -.17]	[-.39, -.20]	[-.29, -.10]	[-.28, -.08]	[-.33, -.13]	[-.08, .09]
Race										
American Indian or Alaska Native	[-.13, .03]	[-.13, .04]	[-.12, .03]	[-.13, .02]	[-.14, .01]	[-.08, .07]	[-.08, .07]	[-.10, .05]	[-.10, .05]	[-.01, .12]
Asian	[-.08, .09]	[-.10, .07]	[-.05, .12]	[-.16, .00]	[-.14, .03]	[-.09, .07]	[-.08, .09]	[-.07, .10]	[-.04, .12]	[-.03, .12]
Native Hawaiian or other Pacific Islander	--	--	--	--	--	--	--	--	--	--
Black or African American	[-.15, .01]	[-.12, .05]	[-.15, .02]	[-.16, .00]	[-.13, .03]	[-.11, .05]	[-.20, -.04]	[-.16, .00]	[-.16, .01]	[.02, .16]
More than one race	[-.06, .10]	[-.08, .09]	[-.11, .05]	[-.08, .07]	[-.04, .12]	[-.07, .08]	[-.05, .11]	[-.03, .14]	[-.08, .08]	[-.08, .06]
Other	[-.06, .11]	[-.12, .06]	[-.07, .10]	[-.06, .10]	[-.10, .07]	[-.02, .14]	[-.11, .05]	[-.12, .05]	[-.10, .07]	[-.07, .08]
White	Ref	Ref	Ref	Ref	Ref	Ref	Ref	Ref	Ref	Ref
Ethnicity										
Hispanic/Latino	[-.14, .05]	[-.09, .10]	[-.07, .11]	[-.20, -.03]	[-.14, .04]	[-.20, -.02]	[-.16, .02]	[-.11, .07]	[-.11, .07]	[.05, .21]
Not Hispanic/Latino	Ref	Ref	Ref	Ref	Ref	Ref	Ref	Ref	Ref	Ref
Education	[-.08, .09]	[-.13, .04]	[-010, .06]	[-.10, .06]	[-.10, .07]	[-.11, .04]	[-.02, .15]	[-.07, .10]	[-.06, .10]	[-.14, .01]
Region of residence										
Midwest	[-.07, .12]	[-.09, .11]	[-.06, .13]	[-.16, .02]	[-.17, .02]	[-.14, .04]	[-.12, .07]	[-.12, .07]	[-.09, .10]	[-.10, .06]
South	[-.10, .10]	[-.13, .07]	[-.08, .11]	[-.15, .03]	[-.18, .01]	[-.11, .08]	[-.16, .03]	[-.13, .06]	[-.06, .13]	[-.06, .10]
West	[-.07, .13]	[-.02, .18]	[-.04, .16]	[-.11, .07]	[-.11, .09]	[-.09, .10]	[-.13, .07]	[-.09, .10]	[-.11, .08]	[-.16, .01]
Northeast	Ref	Ref	Ref	Ref	Ref	Ref	Ref	Ref	Ref	Ref
Party identification										
Republican	[-.05, .17]	[-.08, .14]	[-.05, .16]	[.04, .24]	[-.04, .17]	[.01, .21]	[-.06, .15]	[-.09, .12]	[-.04, .17]	[-.08, .10]
Independents	[-.09, .10]	[-.10, .08]	[-.11, .07]	[.00, .16]	[-.04, .13]	[-.06, .10]	[-.08, .09]	[-.05, .13]	[-.08, .09]	[-.13, .02]
Third party	[-.07, .09]	[-.08, .08]	[-.06, .10]	[-.05, .09]	[-.06, .09]	[-.04, .11]	[-.07, .08]	[-.07, .08]	[-.05, .10]	[-.04, .09]
Democratic	Ref	Ref	Ref	Ref	Ref	Ref	Ref	Ref	Ref	Ref
Political orientation	[-.10, ,10]	[-.10, .11]	[-.10, .10]	[-.19, -.01]	[-.13, .06]	[-.18, .00]	[-.12, .07]	[-.11, .08]	[-.16, .03]	[.04, .20]
Health condition	[.06, .24]	[.11, .28]	[.11, .28]	[.06, .22]	[.04, .21]	[.08, .24]	[.05, .21]	[.11, .27]	[.10, .26]	[-.06, .08]
COVID-19 symptom										
Yes	[-.06, .11]	[-.05, .12]	[-.07, .10]	[-.07, .08]	[-.07, .10]	[-.01, .15]	[-.06, .09]	[-.05, .11]	[-.05, .11]	[-.04, .10]
Unsure	[-.08, .08]	[-.09, .07]	[-.11, .05]	[-.08, .07]	[-.07, .09]	[-.02, .13]	[-.06, .10]	[-.07, .09]	[-.04, .12]	[-.06, .08]
No	Ref	Ref	Ref	Ref	Ref	Ref	Ref	Ref	Ref	Ref
COVID-19 testing										
Yes, negative	[-.01, .16]	[-.03, .14]	[.02, .18]	[-.06, .09]	[-.10, .07]	[-.05, .11]	[-.07, .08]	[-.08, .08]	[-.04, .11]	[.00, .14]
Yes, positive	[-.05, .11]	[-.07, .10]	[-.01, .15]	[-.05, .10]	[-.06, .09]	[-.04, .11]	[-.04, .11]	[-.07, .09]	[-.01, .14]	[-.05, .09]
Yes, waiting	[-.05, .11]	[-.06, .11]	[-.04, .11]	[-.04, .10]	[-.06, .09]	[-.03, .11]	[-.08, .07]	[-.07, .09]	[-.05, .10]	[-.10, .04]
No	Ref	Ref	Ref	Ref	Ref	Ref	Ref	Ref	Ref	Ref
Employment status										

Full-time employed by someone else	[-.12, .10]	[-.10, .12]	[-.10, .11]	[-.15, .05]	[-.16, .05]	[-.13, .07]	[-.15, .06]	[-.16, .05]	[-.15, .05]	[.00, .18]
Part-time employed by someone else	[-.06, .12]	[-.06, .12]	[-.07, .10]	[-.05, .11]	[-.10, .07]	[-.11, .05]	[-.05, .11]	[-.10, .06]	[-.11, .05]	[-.01, .14]
Full-time self-employed	[-.03, .16]	[-.03, .17]	[-.06, .13]	[-.03, .14]	[-.06, .11]	[-.08, .08]	[-.04, .13]	[-.05, .12]	[-.08, .09]	[.03, .18]
Laid off, furloughed, or otherwise unemployed due to COVID-19	[-.03, .15]	[-.06, .12]	[-.05, .12]	[-.08, .07]	[-.10, .06]	[-.08, .08]	[-.07, .10]	[-.11, .07]	[-.08, .09]	[-.01, .14]
Unemployed prior to COVID-19	Ref	Ref	Ref	Ref	Ref	Ref	Ref	Ref	Ref	Ref

Note. PTG = Posttraumatic growth. Ref = Reference group.

Table S3. 95% Confidence Interval for Standardized Coefficients from Regression of Mental Health Outcomes on Psychosocial and Behavioral Predictors.

	**Wave 1**			**Wave 2**			**Wave 3**			
	**Stress**	**Depression**	**Anxiety**	**Stress**	**Depression**	**Anxiety**	**Stress**	**Depression**	**Anxiety**	**PTG**
Perceived health risk	[-.02, .18]	[-.09, .12]	[.02, .22]	[-.05, .13]	[-.01, .18]	[.01, .19]	[-.10, .10]	[-.11, .09]	[-.04, .15]	[.03, .20]
Perceived financial risk	[.01, .20]	[-.01, .18]	[-.06, .12]	[.01, .19]	[-.02, .17]	[-.03, .15]	[.03, .22]	[.00, .20]	[-.04, .15]	[-.15, .01]
Rumination	[.05, .30]	[.06, .32]	[-.03, .23]	[.04, .27]	[.01, .25]	[-.04, .19]	[.10, .35]	[.03, .28]	[-.01, .24]	[-.07, .15]
Co-rumination	[.01, .27]	[-.02, .24]	[-.01, .24]	[.13, .37]	[.07, .32]	[.07, .31]	[-.06, .19]	[-.03, .23]	[-.06, .19]	[-.12, .10]
Perceived social support	[-.18, .01]	[-.19, .00]	[-.11, .08]	[-.13, .03]	[-.19, -.02]	[-.10, .07]	[-.21, -.03]	[-.22, -.04]	[-.12, .05]	[.09, .24]
Perceived social strain	[.14, .32]	[.13, .32]	[.19, .37]	[.20, .37]	[.18, .36]	[.24, .41]	[.26, .44]	[.25, .43]	[.30, .48]	[.00, .16]
Adherence to national guidelines	[-.19, -.01]	[-.14, .05]	[-.19, -.01]	[-.21, -.04]	[-.16, .02]	[-.14, .02]	[-.20, -.03]	[-.15, .03]	[-.16, .01]	[-.06, .09]
Forward-focused coping strategy	[-.24, .00]	[-.39, -.14]	[-.20, .03]	[-.22, -.01]	[-.32, -.09]	[-.16, .06]	[-.17, .06]	[-.32, -.08]	[-.19, .05]	[.33, .53]
Trauma-focused coping strategy	[-.02, .22]	[.02, .27]	[.00, .23]	[-.08, .15]	[-.06, .18]	[-.12, .11]	[-.06, .19]	[-.03, .22]	[-.06, .19]	[.10, .31]

Note. PTG = Posttraumatic growth.
